# The provision of epidural analgesia during labor according to maternal birthplace: a Norwegian register study

**DOI:** 10.1186/s12884-020-03021-8

**Published:** 2020-05-26

**Authors:** Åsa Henning Waldum, Anne Flem Jacobsen, Mirjam Lukasse, Anne Cathrine Staff, Ragnhild Sørum Falk, Siri Vangen, Ingvil Krarup Sørbye

**Affiliations:** 1 Division of Obstetrics and Gynaecology, Oslo University Hospital, Sognsvannsveien 20, 0372 Oslo, Norway; 2 Institute of Clinical Medicine, University of Oslo, Oslo, Norway; 3 Institute of Health Sciences, Oslo Metropolitan University, Oslo, Norway; 4 Institute of Health and Social Sciences, University of South-Eastern Norway, Campus Vestfold, Borre, Norway; 5 Oslo Centre for Biostatistics and Epidemiology, Oslo University Hospital, Oslo, Norway; 6 Norwegian National Advisory Unit on Women’s Health, Oslo University Hospital, Oslo, Norway

**Keywords:** Analgesia obstetric, Delivery obstetric, Immigration, Socioeconomic factors, Decision making

## Abstract

**Background:**

The provision of epidural analgesia during labor is ideally a shared decision between the woman and her health care provider. However, immigrant characteristics such as maternal birthplace could affect decision-making and thus access to pain relief. We aimed to assess disparities in the provision of epidural analgesia in planned vaginal birth according to maternal region of birth.

**Methods:**

We performed a nation-wide register study of 842,496 live-born singleton deliveries in Norway between 2000 and 2015. Maternal birthplace was categorized according to the Global Burden of Disease framework. The provision of epidural analgesia was compared in regression models stratified by parity and mode of delivery.

**Results:**

Compared to native-born women, primiparous women from Latin America/Caribbean countries with an instrumental vaginal delivery were most likely to be provided epidural analgesia (OR 2.12, 95%CI 1.69–2.66), whilst multiparous women from Sub-Saharan Africa with a spontaneous vaginal delivery were least likely to be provided epidural analgesia (OR 0.42, 95% C 0.39–0.44). Longer residence time was associated with a higher likelihood of being provided analgesia, whereas effects of maternal education varied by Global Burden of Disease group.

**Conclusions:**

Disparities in the likelihood of being provided epidural analgesia were observed by maternal birthplace. Further studies are needed to consider whether the identified disparities represent women’s own preferences or if they are the result of heterogeneous access to analgesia during labor.

## Background

Women have always sought to relieve pain during labor. The most common methods of analgesia during labor in high-income settings are regional analgesic methods [1]. These include both epidural analgesia, primarily initiated in the first stage of labor; and pudendal analgesia, provided during the second stage of labor [2]. Epidural analgesia is by far the most common method. The provision of analgesia is associated with a positive childbirth experience [3]. However, several factors might influence the need for labor analgesia. Primiparous women and women undergoing instrumental vaginal delivery (vacuum and/or forceps extraction) have an increased need for analgesia compared to multiparous women and women giving birth spontaneously [4].

Ideally, the provision of pain relief during delivery is a shared decision between the healthcare professional and the woman [1, 5]. Shared decision-making includes the patient’s preferences and the healthcare professional’s knowledge about the benefits and risks of each option [5–7]. Health system characteristics, for example the level of training and skills in obstetrics, may influence the health care staff’s provision of analgesia. The provision of regional analgesic methods during labor requires more information and cooperation between the health care staff and the woman, compared to other types of pain relief [8]. In contrast, pain relief in a planned cesarean delivery is not prone to a shared decision, as protocols are mainly standardized.

Women in reproductive age across most European countries are becoming more ethnically diverse [9]. Previous studies have shown an increased risk of substandard care and poor maternal and neonatal outcome among certain immigrant groups [10–13]. Although women’s desires for pain relief vary, a shared decision might be affected by immigrant characteristics. The woman’s birthplace, health literacy, and residence time in a new country could affect the rate of analgesia during labor. A few Norwegian studies have explored the association between maternal birthplace and the provision of epidural analgesia. These have suggested that Somali and Pakistani migrant women were provided less pain relief during labor than women without migration background. Little is known about women from other regions of the world [8, 10]. Thus, in this study, we aimed to assess the provision of epidural analgesia according to maternal birthplace during planned vaginal birth. Norway is a suitable setting for assessing disparities in epidural analgesia, due to a national compulsory registration of all deliveries and a universal public health care system including free of charge pregnancy ante- and perinatal care.

## Methods

### Study design and population

In Norway most childbirths take place inside the public health care system at hospitals and very few women give birth at home. We linked data from the Medical Birth Register of Norway (MBRN) [14, 15] to information from Statistics Norway about maternal birthplace, maternal education, year of arrival to Norway and reason for immigration (for immigrants only). The MBRN is a national registry with mandatory reporting of all pregnancies ending after 12 complete weeks of gestation. We initially extracted information on all vaginal births in Norway between January 2000 and December 2015. We excluded neonates with a birth weight < 500 g and/or gestational age < 23 weeks. To avoid misclassifications, we excluded deliveries with z-scores (birth weight for gestational week and sex) > ±4 standard deviations. We also excluded abortions and intrauterine fetal death, births with missing maternal birthplace, multiple births and births outside institutions as these women were not eligible for analgesia. Finally, we excluded planned cesarean deliveries, as analgesia is routinely given to these women during surgery (Supplementary Flowchart).

### Variables

The primary outcome was the provision of epidural analgesia. The MBRN registers all types of analgesia provided during labor. Each woman could utilize more than one method of analgesia. The case reporting form contains of tick-boxes for each type of analgesia, which the attending midwife fills out after delivery.

The main exposure was the mother’s own birthplace, as registered by Statistics Norway. For immigrants, the mother’s country of birth was categorized into region of birth according to the Global Burden of Disease (GBD) framework [16]. All high-income countries were grouped into the category “high-income countries”. Furthermore, middle- and low-income countries were categorized into the following GBD regions: “Europe/Central Asia”, “Sub-Saharan Africa”, “North Africa/Middle East”, “South Asia”, “East Asia/Pacific”, or “Latin America/Caribbean”. Norwegian-born women were categorized as “native-born”.

Independent variables were pre-defined and selected based on their potential association with the outcome and exposure according to previous literature. Maternal age at delivery was categorized as < 20, 20–34, 35–40 or ≥ 40 years. Marital status was categorized as married/cohabiting or not. Paternal birthplace was categorized as native-born or not. Maternal education was defined as completed years of education and categorized as lower (≤10 years), middle (11–13 years), or higher (> 13 years). Year of delivery was categorized into 4 periods (2000–2003, 2004–2007, 2008–2011 or 2012–2015). For immigrants we further included maternal residence time in Norway by subtracting the year of first arrival in Norway from the year of delivery, and categorized residence time as < 2 years, 2–10 years, or > 10 years, and reason for immigration (refugee, labor/education, family reunification or other).

Parity was dichotomized as primiparous or multiparous. Mode of delivery was categorized as spontaneous, instrumental (forceps and/or vacuum extraction) or emergency cesarean delivery. Previous cesarean delivery was noted for the multiparous women. Gestational age was categorized as < 37 weeks, 37–41 weeks, or ≥ 42 completed weeks and birth weight into < 2500 g (g), 2500–3999 g, or ≥ 4000 g. Epidural, spinal and pudendal analgesia were noted as utilized (y/n), as were nitrous oxide, local anesthetics and opiates. The size of the obstetric department was pre-categorized by the MBRN as numbers of births per year (1–499, 500–1499, 1500–2999, or ≥ 3000).

### Statistical analyses

Descriptive statistics are presented as frequencies and proportions according to maternal birthplace. The provision of epidural analgesia is presented as proportions within each group of maternal birthplaces stratified by mode of delivery and parity.

The association between epidural analgesia and birthplace was investigated with logistic regression analyses. Women born in Norway were defined as the reference group. Both crude and adjusted odds ratios (OR) with 95% confidence intervals (CI) are presented. Adjustments were made for maternal age at delivery, marital status, education, birth weight, year of childbirth, and size of obstetric department. Gestational age was not included in the regression analyses due to its strong correlation to birth weight. For multiparous women, we adjusted for number of previous births and previous cesarean delivery.

We performed stratified analyses by mode of delivery. Since there was an interaction between maternal birthplace and parity, we also stratified for parity. Further, interaction between maternal birthplace and the adjusting variables were explored by entering the interaction terms, one at a time, into the model. Interactions with *p* < 0.001 are reported in the text and presented graphically.

We performed sensitivity analysis to explore the impact of residence time in Norway, where we included residence time in the regression models. In these analyses, we used women born in high-income countries as reference.

Due to the large sample size, we considered an association with p-value of <.001 as statistically significant. We conducted all analyses using SPSS version 25 (IBM Corp., Armonk, NY, USA) and Stata (StataCorp. 2017. *Stata Statistical Software: Release 15.* College Station, TX: StatCorp LLC).

### Ethics approval and consent to participate

The study was approved by the Regional Committees for Medical and Health Research Ethics South East Norway in 2017 (reference 2016/417/REK) including waiver of participant’s individual consent.

## Results

The final study population included 842,496 deliveries during the 16-year period. Maternal demographic and obstetric characteristics by maternal birthplace are presented in Tables 1 and 2. Immigrant women accounted for 21% of the births (*n* = 175,038). The two largest immigrant groups included women born in high-income countries and Europe/Central Asia, whilst the smallest immigrant group included women born in Latin America/Caribbean (3%) (Supplementary Table 1). Compared to native-born women, a higher proportion of immigrants had lower education, as well as a previous cesarean delivery. The Sub-Saharan African group had the lowest proportion of primiparous women (31.6%). The large obstetric departments had the highest rate of immigrant deliveries. The number of deliveries increased over the study years for all immigrant groups in contrast to the declining birth number in the native-born (in Norway) group. Residence time and reasons for migration varied between GBD groups, and according to known historic migration patterns (Table 1). The proportion of instrumental vaginal delivery was highest among women from East Asia/Pacific (12.4%) compared to 9.4% among native-born women, while women from Sub-Saharan Africa had 17.3% emergency cesarean deliveries compared to 9.5% among native-born women. Sub-Saharan African women had the highest proportion on pregnancies ≥42 weeks of gestational age while native-born women had the highest proportion of newborn weighing ≥4000 g. Nitrous oxide was the most common analgesia method (40.9%), followed by local anesthesia (30.8%), epidural analgesia (30.0%), spinal analgesia (6.4%), opiates (4.7%), and pudendal analgesia (2.4%) (Table 2).

**Table 1:** Demographic characteristics by maternal birthplace, *n* = 842,496 women

	Native-born		Immigrants													
	Norway		High-income		Europe/Central Asia		Sub-Saharan Africa		North Africa/ Middle East		South Asia		East Asia/ Pacific		Latin America/ Caribbean	
	n	**%**	n	**%**	n	**%**	n	**%**	n	**%**	n	**%**	n	**%**	n	**%**
	667,458	79.2	41,450	4.9	41,185	4.9	23,499	2.8	26,735	3.2	22,585	1.4	25,107	3.0	5477	0.7
**Maternal age (years)**																
< 20	15,358	2.3	414	1.0	693	1.7	594	2.5	658	2.5	127	1.1	280	1.1	167	3.0
20–34	540,595	81.0	30,545	73.7	34,646	84.1	18,692	79.5	21,556	80.6	9974	86.1	20,052	79.9	4207	76.8
35–40	102,249	15.3	9487	22.9	5273	12.8	3637	15.5	3946	14.8	1330	11.5	4278	17.0	992	18.1
> 40	9256	1.4	1004	2.4	573	1.4	576	2.5	575	2.2	154	1.3	497	2.0	111	2.0
**Married/Cohabiting**	618,042	92.6	39,156	94.5	39,115	95.0	17,744	75.5	25,107	93.9	11,113	95.9	23,284	92.8	4899	89.4
**Paternal birthplace**																
Native-born	609,302	91.3	27,540	66.4	9612	23.3	2253	9.6	1532	5.7	2553	22.0	12,281	48.9	4266	77.9
Immigrant	49,586	7.4	13,090	31.6	30,321	73.6	17,689	75.3	24,014	89.8	8775	75.7	12,132	48.3	1061	19.4
Missing	8570	1.3	820	2.0	1252	3.0	3557	15.1	1189	4.4	257	2.2	694	2.8	150	2.7
**Maternal education** (years)																
Lower (≤10)	92,166	13.8	3723	9.0	6630	16.1	9944	42.3	10,215	38.2	3971	34.3	7644	30.4	1331	24.3
Middle (11–13)	209,538	31.4	8580	20.7	9161	22.2	3745	15.9	4786	17.9	2089	18.0	5617	22.4	1239	22.6
Higher (> 13)	364,809	54.7	23,751	57.3	16,921	41.1	3267	13.9	5047	18.9	2915	25.2	7550	30.1	2019	36.9
missing	945	0.1	5396	13.0	8473	20.6	6543	27.8	6687	25.0	2610	22.5	4296	17.1	888	16.2
**Size of obstetric department (**births/year)																
1–499	74,040	11.1	2912	7.0	3806	9.2	1865	7.9	1452	5.4	182	1.6	1923	7.7	389	7.1
500–1499	155,308	23.3	6187	14.9	7507	18.2	3473	14.8	3023	11.3	443	3.8	3954	15.7	906	16.5
1500–2999	175,128	26.2	12,710	30.7	12,053	29.3	6106	26.0	8213	30.7	2825	24.4	6400	25.5	1484	27.1
> 3000	262,982	39.4	19,641	47.4	17,819	43.3	12,055	51.3	14,047	52.5	8135	70.2	12,830	51.1	2698	49.3
**Year of delivery**																
2000–2003	161,292	26.6	18,908	21.2	4949	11.2	3393	13.7	5193	18.0	3109	20.6	5387	19.7	943	15.4
2004–2007	155,216	25.6	20,784	23.3	7092	16.0	5087	20.5	6743	23.4	3613	23.9	6236	22.8	1347	22.0
2008–2011	153,243	25.2	24,158	27.1	13,079	29.6	7330	29.6	7889	27.4	4005	26.5	7283	26.7	1823	29.8
2012–2015	137,390	22.6	25,173	28.3	19,095	43.2	8965	36.2	8964	31.1	4393	29.1	8406	30.8	2008	32.8
**Residence time (years)**^1^																
< 2			5124	12.4	12,095	29.4	5928	25.2	6926	25.9	2037	17.6	5321	21.2	1173	21.4
2–10			19,847	47.9	23,709	57.6	13,786	58.7	14,245	53.3	5334	46.0	13,655	54.4	2507	45.8
> 10			6142	14.8	5281	12.8	3216	13.7	5475	20.5	3641	31.4	5452	21.7	569	10.4
Missing			10,337	24.9	100	0.2	569	2.4	89	0.3	573	5.0	679	2.7	1228	22.4
**Reason for immigration**^1^																
Refugee			101	0.2	6621	16.1	11,784	50.2	5786	21.6	186	1.6	1662	6.6	93	1.7
Labor/education			6703	16.2	15,056	36.6	952	4.1	354	1.3	428	3.7	3195	12.7	613	11.2
Family reunification			6529	15.8	18,199	44.2	9425	40.1	18,295	68.4	7938	68.5	16,718	66.6	3304	60.3
Other			12,549	30.3	274	0.7	252	1.1	253	1.0	99	0.9	168	0.7	49	0.9
Missing			15,568	37.6	1035	2.5	1086	4.6	2047	7.7	2934	25.3	3364	13.4	1418	25.9

^1^Immigrants only

**Table 2:** Obstetric characteristics by maternal birthplace, n = 842,496 women

	Native-born		Immigrants													
	Norway		High-income		Europe/Central Asia		Sub-Saharan Africa		North Africa/Middle East		South Asia		East Asia/ Pacific		Latin America/ Caribbean	
	n	**%**	n	**%**	n	**%**	n	**%**	n	**%**	n	**%**	n	**%**	n	**%**
	667,458	79.2	41,450	4.9	41,185	4.9	23,499	2.8	26,735	3.2	22,585	1.4	25,107	3.0	5477	0.7
**Primiparous**	286,766	43.0	19,082	46.0	20,468	49.7	7434	31.6	9751	36.5	4148	35.8	11,223	44.7	2778	50.7
**Mode of delivery**																
Spontaneous vaginal	541,515	81.1	32,118	77.5	32,343	78.5	17,391	74.0	21,222	79.4	9007	77.7	18,173	72.4	3927	71.7
Instrumental vaginal	62,722	9.4	4761	11.5	4750	11.5	2037	8.7	2627	9.8	1212	10.5	3116	12.4	589	10.8
Emergency cesarean section	63,221	9.5	4571	11.0	4092	9.9	4071	17.3	2886	10.8	1366	11.8	3818	15.2	961	15.7
**Previous cesarean delivery**^**1**^	42,145	11.1	2759	12.3	2295	11.1	2833	17.6	2158	12.7	1229	16.5	1978	14.2	528	19.6
**Gestational age** (weeks)																
< 37	33,335	5.0	1928	4.7	1755	4.3	1144	4.9	1332	5.0	769	6.6	1724	6.9	305	5.6
37–41	590,202	88.4	36,913	89.1	36,920	89.6	19,996	85.1	24,063	90.0	10,361	89.4	22,649	90.2	4876	89.0
≥ 42	43,921	6.6	2609	6.3	2510	6.1	2359	10.0	1340	5.0	455	3.9	734	2.9	296	5.4
**Birth weight** (gram)																
< 2500	21,084	3.2	1280	3.1	1170	2.8	1100	4.7	1021	3.8	786	6.8	1162	4.6	212	3.9
2500–3999	504,827	75.6	32,584	78.6	32,992	80.1	19,437	82.7	22,489	84–1	9962	86.0	21,408	85.3	4525	82.6
> 4000	141,547	21.2	7586	18.3	7023	17.1	2962	12.6	3225	12.1	837	7.2	2537	10.1	740	13.5
**Epidural analgesia**^**2**^	199,822	29.9	13,970	33.7	13,898	33.7	5161	22.0	7267	27.2	3188	27.5	6755	26.9	2498	45.6
**Spinal analgesia**^**2**^	41,312	6.2	2794	6.7	2451	6.0	2289	9.7	1742	6.5	829	7.2	2202	8.8	519	9.5
**Pudendal analgesia**^**2**^	16,379	2.5	1179	2.8	908	2.2	383	1.6	497	1.9	240	2.1	566	2.3	144	2.6
**Nitrous Oxide**^**2**^	281,531	42.2	16,161	39.0	16,131	39.2	7569	32.2	9161	34.3	3496	30.2	8611	34.3	2110	38.5
**Local anesthesia**^**2**^	207,465	31.1	12,852	31.0	11,981	29.1	6449	27.4	7052	26.4	3696	31.9	8231	32.8	1442	26.3
**Opiates**^**2**^	32,861	4.9	1628	3.9	1264	3.1	659	2.8	1285	4.8	517	4.5	1113	4.4	203	3.7

^1^Multiparous women only. ^2^Multiple analgesia modalities may have been used

Across all GBD groups, primiparous women were - as expected - provided epidural analgesia more often, when compared to multiparous women (Table 3). Similarly, women with instrumental delivery were more often provided epidural analgesia compared to women with spontaneous delivery. The lowest provision of epidural analgesia was observed among multiparous women from Sub-Saharan Africa delivering spontaneously (9%). The highest provision of epidural analgesia was observed in primiparous Latin America/Caribbean women with an instrumental vaginal delivery (78%). Among women delivered with emergency cesarean section, women from Latin America/Caribbean had the highest epidural analgesia rate (Table 3).

**Table 3:** Epidural analgesia provision by maternal birthplace, mode of delivery and parity

	Spontaneous Vaginal Delivery Epidural analgesia			Instrumental Vaginal Delivery* Epidural analgesia			Emergency Cesarean Delivery Epidural analgesia		
	n	%	95% CI	n	%	95% CI	n	%	95% CI
**Primiparous**	**94,093**	**37.8**	**37.6–38.0**	**38,580**	**61.7**	**61.3–62.0**	**27,126**	**53.8**	**53.4–54.2**
Norway	75,389	37.6	37.4–37.8	29,556	61.1	60.7–61.5	20,199	53.4	52.9–53.9
High-income	4973	40.0	39.1–40.9	2392	64.8	63.2–66.3	1729	58.5	56.7–60.2
Europe / Central Asia	5777	41.2	40.4–42.0	2458	65.2	63.7–66.8	1546	57.7	55.8–59.6
Sub-Saharan Africa	1418	33.8	32.4–35.3	734	57.5	54.8–60.3	937	47.7	45.5–50.0
North Africa / Middle East	2541	39.4	38.2–40.6	1230	66.5	64.3–68.7	741	51.2	48.6–53.8
South Asia	998	38.1	36.2–40.0	555	65.6	62.3–68.8	326	47.9	44.1–51.8
East Asia /Pacific	2085	31.0	29.9–32.2	1298	56.6	54.5–58.6	1232	55.6	53.5–57.7
Latin America/ Caribbean	912	53.8	51.4–56.2	357	78.3	74.2–82.0	416	66.5	62.6–70.1
**Multiparous**	**72,612**	**17.0**	**16.9–17.1**	**9598**	**49.9**	**49.2–50.6**	**10,550**	**30.5**	**30.0–31.0**
Norway	59,723	17.5	17.4–17.6	7256	50.6	49.8–51.4	7699	30.4	29.8–30.9
High-income	3716	18.9	18.3–19.4	599	56.1	53.0–59.1	561	34.8	32.5–37.2
Europe / Central Asia	3104	16.9	16.4–17.5	518	52.7	49.6–55.9	495	35.0	32.5–37.6
Sub-Saharan Africa	1224	9.3	8.8–9.8	279	36.7	33.2–40.2	569	27.0	25.1–28.9
North Africa / Middle East	2020	13.7	13.1–14.2	356	45.8	42.2–49.3	379	26.3	24.1–28.7
South Asia	942	14.8	13.9–15.6	176	48.1	42.9–53.3	191	27.8	24.5–31.4
East Asia /Pacific	1282	11.2	10.6–11.8	328	39.9	36.5–43.3	530	33.0	30.7–35.4
Latin America /Caribbean	601	26.9	25.1–28.8	86	64.7	55.9–72.7	126	37.6	32.4–43.0

*CI* confidence interval *includes deliveries by vacuum and/or forceps extraction

The regression analysis showed heterogeneity in the likelihood of providing epidural analgesia by maternal birthplace. Primiparous women with spontaneous and instrumental vaginal delivery and women born in Sub-Saharan Africa and East Asia/Pacific had lower odds of being provided epidural analgesia compared to native-born women. Women from Latin America/Caribbean were more likely to be provided analgesia. Women born in Sub-Saharan Africa were least likely to be provided epidural analgesia, if they were subsequently delivered by emergency cesarean section, while this was the opposite in women born in Latin America/Caribbean. For multiparous women in all modes of delivery, women from Sub-Saharan Africa had the lowest odds of being provided epidural analgesia, while women from Latin America/Caribbean had the highest odds (Table 4). In addition, we observed a linear effect of time during the study period; the provision of epidural analgesia increased by 3.5–5% each year.

**Table 4:** Epidural analgesia provision by maternal birthplace, stratified by parity

**Spontaneous vaginal delivery**	Primiparous women N 248661								Multiparous women** N 427035							
Global Burden of Disease	Crude OR	95% CI		*P*-value	Adjusted OR*	95% CI		*P-*value	Crude OR	95% CI		*P*-value	Adjusted OR*	95% CI		*P-*value
Norway	Ref.				Ref.				Ref.				Ref.			
High-income	1.11	1.07	1.15	< 0.001	1.09	1.05	1.13	< 0.001	1.10	1.06	1.14	< 0.001	1.04	1.00	1.08	0.028
Europe/Central Asia	1.16	1.12	1.20	< 0.001	1.07	1.03	1.01	< 0.001	0.96	0.92	1.00	0.047	0.83	0.80	0.87	< 0.001
Sub-Saharan Africa	0.85	0.80	0.90	< 0.001	0.74	0.69	0.79	< 0.001	0.48	0.45	0.51	< 0.001	0.42	0.39	0.44	< 0.001
North Africa/Middle East	1.08	1.02	1.13	0.004	1.01	0.96	1.06	0.772	0.75	0.71	0.78	< 0.001	0.68	0.65	0.72	< 0.001
South Asia	1.02	0.94	1.10	0.624	1.01	0.92	1.09	0.963	0.82	0.76	0.87	< 0.001	0.72	0.67	0.78	< 0.001
East Asia/Pacific	0.75	0.71	0.79	< 0.001	0.74	0.70	0.78	< 0.001	0.59	0.56	0.63	< 0.001	0.53	0.49	0.56	< 0.001
Latin America/Caribbean	1.93	1.75	2.13	< 0.001	1.83	1.66	2.02	< 0.001	1.74	1.58	1.91	< 0.001	1.44	1.31	1.59	< 0.001
**Instrumental vaginal delivery**	**Primiparous women** **N 62565**								Multiparous women** **N 19249**							
**Global Burden of Disease**	Crude OR	95% CI		*P*-value	Adjusted OR*	95% CI		*P-*value	Crude OR	95% CI		*P*-value	Adjusted OR*	95% CI		*P-*value
Norway	Ref.				Ref.				Ref.				Ref.			
High-income	1.17	1.09	1.26	< 0.001	1.12	1.04	1.20	0.003	1.25	1.10	1.41	0.001	1.12	0.98	1.28	0.084
Europe/Central Asia	1.20	1.12	1.28	< 0.001	1.09	1.01	1.17	0.025	1.09	0.96	1.24	0.194	0.98	0.86	1.13	0.828
Sub-Saharan Africa	0.86	0.77	0.97	0.01	0.78	0.69	0.88	< 0.001	0.57	0.49	0.66	< 0.001	0.52	0.44	0.62	< 0.001
North Africa/Middle East	1.27	1.15	1.40	< 0.001	1.18	1.06	1.30	0.002	0.82	0.71	0.95	0.009	0.82	0.70	0.97	0.018
South Asia	1.22	1.05	1.40	0.008	1.14	0.98	1.32	0.089	0.90	0.74	1.11	0.341	0.76	0.61	0.95	0.016
East Asia/Pacific	0.83	0.76	0.90	< 0.001	0.80	0.73	0.87	< 0.001	0.65	0.56	0.75	< 0.001	0.61	0.52	0.71	< 0.001
Latin America/Caribbean	2.30	1.84	2.87	< 0.001	2.12	1.69	2.66	< 0.001	1.79	1.25	2.55	0.001	1.58	1.09	2.28	0.016
**Emergency cesarean delivery**	**Primiparous women** **N 50424**								Multiparous women** **N 34562**							
**Global Burden of Disease**	Crude OR	95% CI		*P*-value	Adjusted OR*	95% CI		*P-*value	Crude OR	95% CI		*P*-value	Adjusted OR*	95% CI		*P-*value
Norway	Ref.				Ref.				Ref.				Ref.			
High-income	1.23	1.14	1.33	< 0.001	1.14	1.05	1.24	0.002	1.22	1.10	1.36	< 0.001	1.07	0.95	1.20	0.254
Europe/Central Asia	1.19	1.10	1.29	< 0.001	1.03	0.94	1.13	0.503	1.24	1.10	1.38	< 0.001	1.11	0.98	1.25	0.103
Sub-Saharan Africa	0.80	0.73	0.87	< 0.001	0.82	0.74	0.91	< 0.001	0.85	0.77	0.94	0.001	0.80	0.71	0.90	< 0.001
North Africa/Middle East	0.92	0.83	1.02	0.109	0.95	0.85	1.07	0.402	0.82	0.73	0.93	0.001	0.81	0.71	0.92	0.002
South Asia	0.81	0.69	0.94	0.005	0.90	0.76	1.07	0.225	0.89	0.75	1.05	0.158	0.92	0.77	1.11	0.375
East Asia/Pacific	1.10	1.01	1.20	0.036	1.06	0.97	1.17	0.215	1.13	1.02	1.26	0.023	1.07	0.95	1.21	0.241
Latin America/Caribbean	1.73	1.47	2.05	< 0.001	1.68	1.40	2.01	< 0.001	1.38	1.11	1.73	0.004	1.21	0.95	1.54	0.119

*Adjusted for: age at delivery (< 20, 20–34, ≥35 years), marital status (married/cohabiting y/n), maternal education (lower, middle, higher, missing), birth weight, year of childbirth and size of obstetric department. **In multiparous women, also for parity (1, 2, 3, 4+ births) and previous cesarean delivery (y/n). *GBD* Global Burden of Disease; *OR* Odds ratio; *CI* Confidence interval

**Fig. 1:**
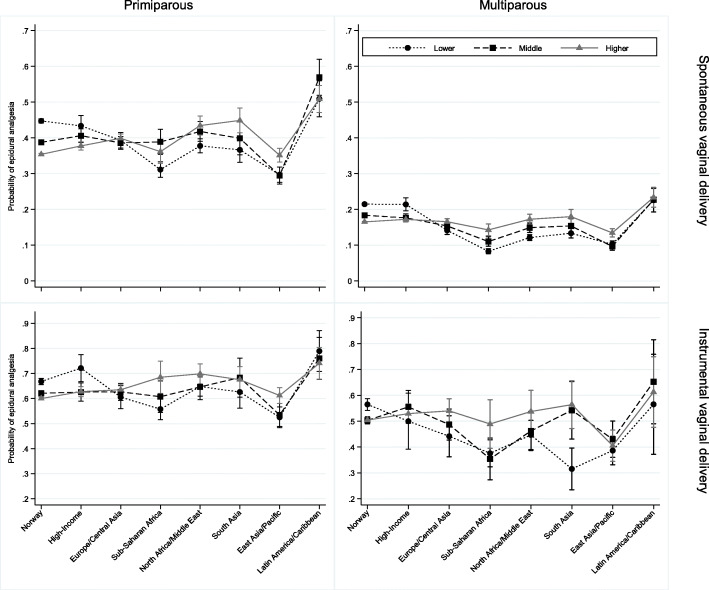
Probability of epidural analgesia provision by maternal region of birth according to maternal education, stratified by mode of delivery (rows) and parity (columns). Maternal education; lower (≤10 years), middle (11–13 years) and higher (> 13 years). Error bars: 95% confidence intervals

Maternal education modified the effect of maternal birthplace on the likelihood of being provided epidural analgesia. Among native-born women, those with higher education were less likely to be provided epidural analgesia compared to those with lower education. Conversely, among several immigrant groups from medium- or low-income countries (Europe/Central Asia, Sub-Saharan Africa, North Africa/Middle East, South Asia, or East Asia/Pacific), those with higher education were more likely to be provided epidural analgesia compared to those with lower education (Fig. 1).

However, within education categories, the absolute likelihood of being provided epidural analgesia was lower among the majority of immigrants, compared to native-born women. Furthermore, among multiparous women who delivered spontaneously, interaction effects were observed between maternal birthplace and parity and between maternal birthplace and year of childbirth. Among women from Europe/Central Asia, Sub-Saharan Africa, North Africa/Middle East, South Asia, and East Asia/Pacific, the provision of epidural analgesia decreased by increasing parity, while in the other groups, the provision did not differ by number of deliveries (data not shown). For the same immigrant groups, the increasing provision of epidural analgesia by calendar time was more pronounced than in the remaining groups (data not shown). Only one interaction effect was observed among women with emergency cesarean section; previous cesarean section reduced the risk of epidural analgesia in women from East Asia/Pacific.

In sensitivity analyses among immigrants only, residence time slightly affected the association between maternal birthplace and provision of epidural analgesia. Among primiparous women with an instrumental delivery, provision of epidural analgesia was increased by the residence time in Norway (Supplementary Table 2).

Finally, we found an almost 50% reduction of preeclampsia, but stable rates of diabetes over the time period (Supplementary Table 3). Sub analyses for spinal analgesia and pudendal block revealed the same direction of effect (Supplementary Table 4 and 5).

## Discussion

### Summary of evidence

This study, using population-based data from the Norwegian Birth Registry shows that there is a globally significant effect of birthplace on the use of epidural analgesia. We demonstrated some disparities in the provision of epidural analgesia by maternal birthplace. Immigrants from Latin America/Caribbean were consistently more likely to be provided epidural analgesia compared to native-born women. In contrast, the provision of epidural analgesia in immigrants born in low- and middle-income countries varied across maternal birthplace. Compared to native-born women, women born in Sub-Saharan Africa or East Asia/Pacific were less likely to be provided epidural analgesia. Longer residence time in Norway was associated with a higher likelihood of being provided analgesia, whereas effects of maternal education depended on GBD group.

Our results for Sub-Saharan women in Norway confirms findings from Bakken et al. (2015) of the low provision of epidural analgesia among Somali-born immigrants, the largest migrant group from Sub-Saharan Africa in Norway [10]. Regarding South Asian women, we found that primiparous women had similar chances of being provided epidural analgesia as native-born women, whereas multiparous women had a reduced likelihood. This is in line with a previous Norwegian study by Vangen et al. [8], where Pakistani-born women were found to be provided less epidural analgesia regardless of parity.

Our results are likely to have multicausal explanations. Firstly, our result could be influenced by real differences in women’s own wishes and needs. Cultural norms and perceptions of labor pain as well as knowledge of side effects of pain relief could affect women’s choices, even though women’s prenatal analgesia preference does not always match their actual use [17]. However, we found significant variation also in women exposed to an instrumental vaginal delivery, where pain relief is strongly advised by the midwife and obstetrician. Overall, primiparous women had a higher likelihood of being provided epidural analgesia compared to multiparous women, and women with instrumental vaginal delivery had a higher likelihood compared to spontaneous delivery. This was expected, as primiparous women have a longer duration of delivery and more interventions by instrumental deliveries. In addition, instrumental vaginal delivery is more painful than spontaneous vaginal deliveries.Secondly, our results can be explained by pre-migration exposure to health system practices and norms in the home country. We found high provision of epidural analgesia among women born in Latin America/Caribbean region. In line with this, the epidural analgesia rate in Chile and in private health facilities in Brazil is higher than in Norway [18, 19]. Conversely, in low- and middle-income countries, access to epidural analgesia is often suboptimal. In our study we found a low provision of epidural analgesia in women born in Sub-Saharan Africa. Outside tertiary facilities in these women’s home countries management of labor pain often only involves non-pharmacological pain relief [20].

Thirdly, low uptake of pain relief in certain groups could be influenced by suboptimal communication, especially if language barriers were present. A study among Hispanic women in the US found lower provision of epidural analgesia among Hispanic women as compared to whites, and language barriers mediated that difference [21]. Both language barriers and misconceptions about possible pain relief may contribute to a communication barrier between the women and the health care providers [18]. Orejula et al. reported misconceptions about the safety of epidural analgesia in foreign-born women [18]. When language barriers are present, individual support by a laywoman (doula) matched by language and cultural preference of the woman giving birth, has been attempted with the aim of providing translation and advocacy to the woman [22]. A recent Cochrane review supports the use of doula as a resource to foreign-born delivering women in high-income countries [23].

Finally, health literacy and level of education could also have impacted our findings. Women in minority groups have previously reported poorer experience of maternity services [24]. We cannot exclude that lower provision of epidural may be determined by a paternalistic attitude among the healthcare staff towards women of lower socioeducational groups. Higher educated women born in Sub-Saharan Africa, North Africa/Middle East or South Asia were more likely to be provided epidural analgesia, compared to those with lower education from the same areas. Furthermore, we found increased provision of epidural analgesia with longer residence in Norway, implying a potential acculturation effect [25]. In our study, Pakistani born women constituted 72% of the South Asian group, which also was the group of women that had the longest residence time in Norway in our study. Longer residence time is associated with improved health literacy, including improved language proficiency, which could strengthen the participation in decision-making. Good language skills could modify a negative impact of ethnicity on the provision of analgesia during delivery [21]. At the same time, increasing familiarity with and knowledge of cultural-specific attitudes might improve the effort and communication skills among health staff themselves.

We also examined pain relief in emergency cesarean deliveries. In Norway, 21% of all cesarean deliveries are due to failure to progress [26], and in these women epidural analgesia is especially useful. However, as we lacked data on indication for emergency cesarean delivery, these results are difficult to interpret.

### Strengths and limitations

Strengths of this study include the use of a large, nation-wide birth cohort with minimal selection bias, including more than 175,000 births among immigrant women. The completeness of the MBRN is close to 100% and misclassifications are believed to be minimal [14, 15]. The linkage with national statistics enabled us to include information on maternal education level and residence time in Norway, as proxy indicators of health literacy and acculturation, respectively. The use of the GBD framework to classify the immigrant population may also be evaluated as strength, as the framework combines geographical and economical aspects of the country of birth.

The study has some limitations. The categorization of different countries into GBD groups may cause a loss of detailed information from particular countries. In addition, we assume that women originating from a particular geographical region share common traits, disregarding the heterogeneity in sociocultural background, religion, attitudes and a selection to migration. We controlled for predefined potential confounders; however, we did not have information on language skills, interpreter use or health literacy. The relative risk of epidural analgesia was based on logistic regression analyses adjusted for potential confounders, assuming additive effects. When investigating the presence of effect modification, using a strict significance level, we found a clear and consistent interaction between the provision of epidural analgesia and education. However, due to the large sample size, we cannot exclude spurious interaction effects and results should be interpreted with caution. In addition, there has been an increased provision of epidural over the study time period. To take into any consideration time-dependent effects, we included year of birth in the regression analysis. Thus, we believe that any bias from time-dependent effects have been adjusted for in the final models.

Other types of pain relief (nitrous oxide, intravenous opiates etc) were outside the scope of this study, however we performed sub analyses for spinal analgesia and pudendal block (Supplementary Table 4 and 5).

Due to the heterogeneity of the immigrant populations, our results cannot necessarily be generalized to other settings. However, in countries with a similar immigration pattern and universal free maternity care, results may be similar. To determine why there are disparities in the provision of epidural analgesia, future studies exploring women’s own perspectives are needed.

## Conclusion

In this study, maternal birthplace was associated with the likelihood of being provided epidural analgesia. Further investigations, quantitative as well as qualitative, may help elucidate reasons for this diversity and provide knowledge about women’s own needs and wishes.

## Supplementary information

**Additional file 1.**

## Data Availability

The anonymous datasets used and/or analyzed during the current study are available from the corresponding author on reasonable request.

## Abbreviations

GBD: Global Burden of Disease
MBRN: Medical Birth Registry of Norway
