# Supplementary material for: The provision of epidural analgesia during labor according to maternal birthplace: a Norwegian register study
Source: BMC Pregnancy Childbirth. 2020 May 26;20:321. doi: 10.1186/s12884-020-03021-8 (PMC7249666; doi:10.1186/s12884-020-03021-8)
Supplement: Supplementary file 1 — Additional file 1. [file 12884_2020_3021_MOESM1_ESM.docx]

Supplementary Material

**The provision of epidural analgesia according to maternal birthplace: a Norwegian register study**

**Waldum ÅH et al.**

**Supplementary Flowchart.** Inclusion and exclusion process


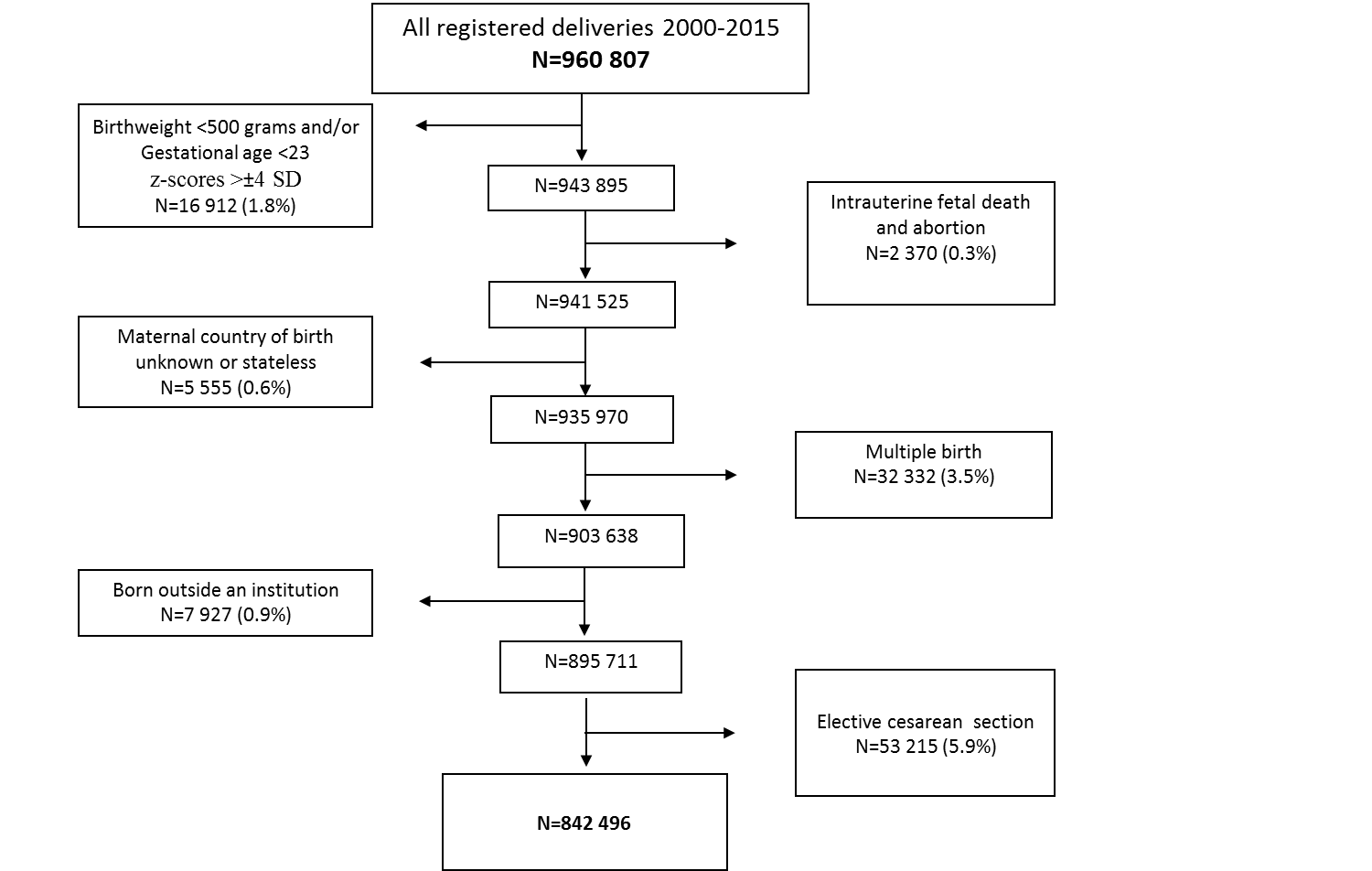


**Supplementary Table 1.** Top 3 countries of maternal birthplace within immigrant group, N=175038

| **Maternal region of birth** | n (%) |
| --- | --- |
| High-income countries, n (% of immigrants)  Sweden, %*  Germany, %*  Denmark, %* | 41450 (23.7)  29.0  11.7  9.3 |
| Europe/Central Asia, n (%)  Poland, %*  Russia, %*  Lithuania, % | 41185 (23.5)  27.6  13.5  12.4 |
| Sub-Saharan Africa, n (%)  Somalia, %*  Eritrea, %*  Ethiopia, %* | 23499 (13.4)  51.2  11.9  10.1 |
| North Africa/Middle East, n (%)  Iraq, %*  Turkey, %*  Afghanistan, %* | 26735 (15.3)  33.5  15.1  12.1 |
| South Asia, n (%)  Pakistan, %*  India, %*  Bangladesh, %* | 11585 (6.6)  72.0  22.7  3.1 |
| East Asia/Pacific, n (%)  Philippines, %*  Thailand, %*  Vietnam, %* | 25107 (14.3)  25.3  22.7  20.8 |
| Latin America/Caribbean, n (%)  Brazil, %*  Colombia, %*  Peru, %* | 5477 (3.1)  32.4  20.5  9.8 |

* percentage within each immigrant group

**Supplementary Table 2.** Associations between residence time and epidural analgesia in primiparous immigrant women with instrumental vaginal delivery, N= 14181

|  | Crude OR | 95% CI | | *P*-value | Adjusted OR* | 95% CI | | *P*-value |
| --- | --- | --- | --- | --- | --- | --- | --- | --- |
| **Global Burden of Disease** |  |  |  |  |  |  |  |  |
| High income | Ref. |  |  |  | Ref. |  |  |  |
| Europe/Central Asia | 1.02 | 0.93 | 1.12 | 0.675 | 1.08 | 0.97 | 1.19 | 0.144 |
| Sub-Saharan Africa | 0.74 | 0.65 | 0.84 | <0.001 | 0.77 | 0.68 | 0.88 | <0.001 |
| North Africa/ Middle East | 1.08 | 0.96 | 1.22 | 0.196 | 1.14 | 1.00 | 1.29 | 0.043 |
| South Asia | 1.04 | 0.89 | 1.21 | 0.648 | 1.07 | 0.91 | 1.25 | 0.429 |
| East Asia/Pacific | 0.71 | 0.64 | 0.79 | <0.001 | 0.74 | 0.66 | 0.82 | <0.001 |
| Latin America/ Caribbean | 1.96 | 1.55 | 2.48 | <0.001 | 2.03 | 1.61 | 2.56 | <0.001 |
| **Residence time** |  |  |  |  |  |  |  |  |
| <2 years | Ref. |  |  |  | Ref. |  |  |  |
| 2-10 years | 1.16 | 1.08 | 1.26 | <0.001 | 1.17 | 1.08 | 1.27 | <0.001 |
| >10 years | 1.43 | 1.28 | 1.61 | <0.001 | 1.46 | 1.30 | 1.64 | <0.001 |
| Missing | 1.36 | 1.18 | 1.56 | <0.001 | 1.29 | 1.11 | 1.51 | 0.001 |

*Adjusted for age at delivery (<20, 20-34, ≥35 years), marital status (married/cohabiting y/n), maternal education (lower, middle, higher, missing), birth weight, year of childbirth and size of obstetric department. GBD: Global Burden of Disease; OR: Odds ratio; CI: Confidence interval.

**Supplementary Table 3.** Rate of maternal disease

|  | Preeclampsia/Eclampsia  % | Diabetes type 1  % |
| --- | --- | --- |
| 2000-2003 | 3.9 | 0.4 |
| 2004-2007 | 3.6 | 0.4 |
| 2008-2011 | 3.2 | 0.4 |
| 2012-2015 | 2.0 | 0.4 |

**Supplementary table 4.** Pudendal block provision by maternal birthplace, stratified by parity

| **Spontaneous vaginal delivery** | **Primiparous women, N 248661** | | | | | | | | | | | | | **Multiparous women, N 427035** | | | | | | | | | | | | | | | | | |
| --- | --- | --- | --- | --- | --- | --- | --- | --- | --- | --- | --- | --- | --- | --- | --- | --- | --- | --- | --- | --- | --- | --- | --- | --- | --- | --- | --- | --- | --- | --- | --- |
| **Global Burden of Disease** | **CrudeOR** | **95% CI** | | | | | **Adj OR*** | | | | **95% CI** | | | | | | **CrudeOR** | **95% CI** | | | | | | **Adj OR**** | | | | **95% CI** | | | |
| Norway | Ref. |  | | | |  | Ref. | | | |  |  | | | | | Ref. |  | | | | |  | Ref. | | | |  | |  | |
| High-income | 1.54 | 1.04 | | | | 1.28 | 1.10 | | | | 0.99 | 1.22 | | | | | 1.21 | 1.09 | | | | | 1.34 | **1.14** | | | | **1.03** | | **1.27** | |
| Europe/Central Asia | 0.83 | 0.75 | | | | 0.94 | 0.91 | | | | 0.81 | 1.03 | | | | | 0.73 | 0.64 | | | | | 0.83 | **0.80** | | | | **0.70** | | **0.92** | |
| Sub-Saharan Africa | 1.05 | 0.87 | | | | 1.26 | 1.14 | | | | 0.94 | 1.37 | | | | | 0.68 | 0.58 | | | | | 0.79 | **0.76** | | | | **0.64** | | **0.90** | |
| North Africa/Middle East | 0.88 | 0.75 | | | | 1.04 | 0.90 | | | | 0.76 | 1.06 | | | | | 0.75 | 0.65 | | | | | 0.87 | **0.78** | | | | **0.67** | | **0.91** | |
| South Asia | 0.89 | 0.70 | | | | 1.14 | 0.85 | | | | 0.66 | 1.09 | | | | | 0.91 | 0.74 | | | | | 1.11 | 0.85 | | | | 0.69 | | 1.04 | |
| East Asia/Pacific | 0.88 | 0.75 | | | | 1.03 | 0.91 | | | | 0.77 | 1.07 | | | | | 0.80 | 0.68 | | | | | 0.93 | **0.81** | | | | **0.69** | | **0.96** | |
| Latin America/Caribbean | 0.88 | 0.65 | | | | 1.21 | 0.89 | | | | 0.65 | 1.22 | | | | | 1.28 | 0.96 | | | | | 1.70 | 1.28 | | | | 0.96 | | 1.70 | |
| **Instrumental vaginal delivery** | **Primiparous women, N 62565** | | | | | | | | | | | | | | **Multiparous women, N 19249** | | | | | | | | | | | | | | | | |
| **Global Burden of Disease** | **CrudeOR** | **95% CI** | | | | | | **Adj OR*** | | | **95% CI** | | | | **CrudeOR** | | | **95% CI** | | | | | | | **Adj OR**** | | | **95% CI** | | | |
| Norway | Ref. |  | | | |  | | Ref. | | |  | |  | | Ref. | | |  | | | | |  | | Ref. | | |  | | |  |
| High-income | 0.99 | | 0.87 | | 1.13 | | | 0.94 | | | 0.82 | | 1.07 | | | 0.99 | | | | 0.77 | | 1.25 | | | 0.99 | | 0.77 | | | | 1.26 |
| Europe/Central Asia | 0.96 | | 0.84 | | 1.09 | | | 0.92 | | | 0.81 | | 1.06 | | | 0.88 | | | | 0.68 | | 1.15 | | | 0.95 | | 0.73 | | | | 1.24 |
| Sub-Saharan Africa | 0.71 | | 0.56 | | 0.91 | | | **0.71** | | | **0.55** | | **0.92** | | | 0.41 | | | | 0.27 | | 0.62 | | | **0.48** | | **0.32** | | | | **0.74** |
| North Africa/Middle East | 0.75 | | 0.61 | | 0.91 | | | **0.72** | | | **0.59** | | **0.89** | | | 0.53 | | | | 0.37 | | 0.76 | | | **0.59** | | **0.40** | | | | **0.86** |
| South Asia | 0.86 | | 0.65 | | 1.14 | | | 0.82 | | | 0.62 | | 1.09 | | | 0.54 | | | | 0.32 | | 0.91 | | | **0.58** | | **0.34** | | | | **0.99** |
| East Asia/Pacific | 0.93 | | 0.78 | | 1.09 | | | 0.91 | | | 0.77 | | 1.08 | | | 1.12 | | | | 0.87 | | 1.45 | | | 1.19 | | 0.91 | | | | 1.56 |
| Latin America/Caribbean | 1.15 | | 0.83 | | 1.61 | | | 1.11 | | | 0.79 | | 1.55 | | | 1.49 | | | | 0.85 | | 2.60 | | | 1.57 | | 0.89 | | | | 2.76 |
| **Emergency cesarean delivery** | **Primiparous women, N 50424** | | | | | | | | | | | | | | | **Multiparous women, N 34562** | | | | | | | | | | | | | | | |
| **Global Burden of Disease** | **CrudeOR** | **95% CI** | | | | | | **Adj OR*** | | **95% CI** | | | | | **CrudeOR** | | | **95% CI** | | | | | | | | **Adj OR**** | | | **95% CI** | | |
| Norway | Ref. |  | | | |  | | Ref. | |  | | |  | | Ref. | | |  | | |  | | | | | Ref. | | |  |  | |
| High-income | 1.09 | | | 0.73 | 1.63 | | | 1.06 | 0.71 | | | | 1.61 | | | 1.08 | | | 0.55 | | 2.13 | | | | | 1.01 | | | 0.51 | | 2.01 |
| Europe/Central Asia | 0.88 | | | 0.55 | 1.40 | | | 0.84 | 0.51 | | | | 1.36 | | | 0.96 | | | 0.45 | | 2.05 | | | | | 0.98 | | | 0.45 | | 2.15 |
| Sub-Saharan Africa | 0.75 | | | 0.42 | 1.35 | | | 0.80 | 0.44 | | | | 1.47 | | | 0.37 | | | 0.14 | | 0.99 | | | | | 0.45 | | | 0.16 | | 1.27 |
| North Africa/Middle East | 0.77 | | | 0.40 | 1.49 | | | 0.85 | 0.43 | | | | 1.68 | | | 0.81 | | | 0.36 | | 1.83 | | | | | 0.91 | | | 0.38 | | 2.16 |
| South Asia | 1.09 | | | 0.49 | 2.46 | | | 1.27 | 0.55 | | | | 2.89 | | | 0.28 | | | 0.04 | | 2.01 | | | | | 0.34 | | | 0.05 | | 2.45 |
| East Asia/Pacific | 0.61 | | | 0.34 | 1.12 | | | 0.66 | 0.36 | | | | 1.22 | | | 1.45 | | | 0.80 | | 2.63 | | | | | 1.51 | | | 0.81 | | 2.83 |
| Latin America/Caribbean | 0.39 | | | 0.10 | 1.58 | | | 0.40 | 0.10 | | | | 1.64 | | | - | | | - | | - | | | | | - | | | - | | - |

*Adjusted for: age at delivery (<20, 20-34, ≥35 years), marital status (married/cohabiting y/n), maternal education (lower, middle, higher, missing), birth weight, year of childbirth and size of obstetric department. **In multiparous women, also adjusted for parity (1, 2, 3, 4+ births) and previous caesarean delivery (y/n).

OR: Odds ratio; CI: Confidence interval.

**Supplementary table 5.** Spinal analgesia provision by maternal birthplace, stratified by parity

| **Spontaneous vaginal delivery** | **Primiparous women, N 248661** | | | | | | | | | **Multiparous women, N 427035** | | | | | |
| --- | --- | --- | --- | --- | --- | --- | --- | --- | --- | --- | --- | --- | --- | --- | --- |
| **Global Burden of Disease** | Crude OR | 95% CI | | | Adj OR* | | 95% CI | | | Crude OR | 95% CI | | Adj OR****** | 95% CI | |
| Norway | Ref. | | | | Ref. | | | | | Ref. | | | Ref. | | |
| High-income | 0.98 | 0.84 | | 1.15 | 0.93 | | 0.79 | | 1.09 | 1.17 | 1.03 | 1.32 | 1.13 | 0.99 | 1.28 |
| Europe/Central Asia | 0.83 | 0.70 | | 0.97 | **0.75** | | **0.63** | | **0.89** | 0.96 | 0.84 | 1.11 | 0.91 | 0.79 | 1.05 |
| Sub-Saharan Africa | 1.20 | 0.93 | | 1.53 | 1.18 | | 0.91 | | 1.52 | 0.64 | 0.53 | 0.79 | **0.66** | **0.53** | **0.81** |
| North Africa/Middle East | 0.81 | 0.64 | | 1.03 | 0.82 | | 0.64 | | 1.04 | 0.67 | 0.56 | 0.81 | **0.71** | **0.58** | **0.86** |
| South Asia | 0.89 | 0.63 | | 1.28 | 0.92 | | 0.64 | | 1.33 | 0.81 | 0.63 | 1.04 | 0.85 | 0.66 | 1.11 |
| East Asia/Pacific | 1.29 | 1.07 | | 1.56 | **1.29** | | **1.06** | | **1.56** | 0.88 | 0.73 | 1.06 | 0.90 | 0.75 | 1.09 |
| Latin America/Caribbean | 1.12 | 0.75 | | 1.66 | 1.08 | | 0.72 | | 1.61 | 1.20 | 0.84 | 1.71 | 1.08 | 0.75 | 1.54 |
| **Instrumental vaginal delivery** | **Primiparous women, N 62565** | | | | | | | | | **Multiparous women, N 19249** | | | | | |
| **Global Burden of Disease** | Crude OR | 95% CI | | | Adj OR* | | 95% CI | | | Crude OR | 95% CI | | Adj OR****** | 95% CI | |
| Norway | Ref. | | | | Ref. | | | | | Ref. | | | Ref. | | |
| High-income | 0.90 | 0.72 | | 1.13 | 0.90 | | 0.71 | | 1.13 | 1.24 | 0.93 | 1.65 | 1.21 | 0.90 | 1.62 |
| Europe/Central Asia | 0.75 | 0.59 | | 0.96 | **0.70** | | **0.54** | | **0.91** | 0.63 | 0.43 | 0.94 | **0.58** | **0.39** | **0.88** |
| Sub-Saharan Africa | 0.67 | 0.43 | | 1.04 | 0.67 | | 0.43 | | 1.04 | 0.73 | 0.48 | 1.11 | 0.71 | 0.45 | 1.12 |
| North Africa/Middle East | 0.80 | 0.57 | | 1.11 | 0.82 | | 0.58 | | 1.15 | 0.74 | 0.49 | 1.12 | 0.73 | 0.47 | 1.13 |
| South Asia | 0.72 | 0.43 | | 1.21 | 0.76 | | 0.45 | | 1.28 | 0.59 | 0.30 | 1.14 | 0.63 | 0.32 | 1.23 |
| East Asia/Pacific | 0.80 | 0.59 | | 1.09 | 0.82 | | 0.60 | | 1.12 | 0.79 | 0.53 | 1.17 | 0.78 | 0.52 | 1.17 |
| Latin America/Caribbean | 0.99 | 0.54 | | 1.81 | 0.98 | | 0.53 | | 1.79 | - | - | - | - | - | - |
| **Emergency cesarean delivery** | **Primiparous women, N 50424** | | | | | | | | | **Multiparous women, N 34562** | | | | | |
| **Global Burden of Disease** | Crude OR | | 95% CI | | | Adj OR* | | 95% CI | | Crude OR | 95% CI | | Adj OR****** | 95% CI | |
| Norway | Ref. | | | | Ref. | | | | | Ref. | | | Ref. | | |
| High-income | 0.87 | 0.80 | | 0.94 | **0.92** | | **0.85** | | **0.99** | 0.94 | 0.85 | 1.05 | 0.98 | 0.88 | 1.08 |
| Europe/Central Asia | 0.91 | 0.84 | | 0.98 | 0.92 | | 0.85 | | 1.00 | 0.87 | 0.79 | 0.97 | 0.89 | 0.80 | 1.00 |
| Sub-Saharan Africa | 0.96 | 0.88 | | 1.05 | 0.96 | | 0.87 | | 1.06 | 0.87 | 0.80 | 0.95 | 0.96 | 0.87 | 1.06 |
| North Africa/Middle East | 0.99 | 0.89 | | 1.10 | 1.01 | | 0.91 | | 1.20 | 0.91 | 0.82 | 1.02 | 0.99 | 0.88 | 1.10 |
| South Asia | 1.01 | 0.87 | | 1.17 | 1.04 | | 0.89 | | 1.22 | 0.92 | 0.79 | 1.08 | 1.00 | 0.86 | 1.17 |
| East Asia/Pacific | 0.89 | 0.81 | | 0.97 | **0.90** | | **0.82** | | **0.99** | 0.92 | 0.83 | 1.02 | 0.98 | 0.88 | 1.09 |
| Latin America/Caribbean | 0.77 | 0.65 | | 0.90 | **0.79** | | **0.67** | | **0.93** | 0.97 | 0.78 | 1.21 | 1.01 | 0.81 | 1.27 |

*Adjusted for: age at delivery (<20, 20-34, ≥35 years), marital status (married/cohabiting y/n), maternal education (lower, middle, higher, missing), birth weight, year of childbirth and size of obstetric department. **In multiparous women, also for parity (1, 2, 3, 4+ births) and previous caesarean delivery (y/n). OR: Odds ratio; CI: Confidence interval.
